# Multiplex PCR to diagnose bloodstream infections in patients after cardiothoracic surgery

**DOI:** 10.1186/2197-425X-3-S1-A884

**Published:** 2015-10-01

**Authors:** K Pilarczyk, P-M Rath, J Steinmann, J Benedik, D Wendt, M Dürbeck, H Jakob, F Dusse

**Affiliations:** West German Heart and Vascular Center Essen, Department of Thoracic and Cardiovascular Surgery, University Hospital Essen, Essen, Germany; Institute of Medical Microbiology, University Hospital Essen, Essen, Germany

## Introduction

Infectious complications and sepsis are major causes of mortality and morbidity after cardiac surgery. Early detection of the causing microorganisms and prompt initiation of adequate antimicrobial therapy are crucial for patient survival. However, blood culture (BC) as the current diagnostic gold standard suffers from low sensitivity, as well as a reporting delay of approximately 48-72 h. LightCycler SeptiFast (SF) is a real-time multiplex polymerase chain reaction test able to detect 25 common pathogens responsible for bloodstream infections within hours.

## Objectives

This single-center retrospective study aimed to compare the performance of SF matched with conventional BC samples and identify predictors for positivity of SF in patients after cardiothoracic surgery.

## Methods

Overall, 279 blood samples from 168 individuals with suspected bloodstream infection were analyzed by both, SF and BC. Receiver operating characteristic (ROC) curves were generated to determine the accuracy of clinical and laboratory information for the prediction of positive SF results.

## Results

Excluding results attributable to contaminants, 14.7% (n = 41) of the blood samples were positive using SF and 17.2% (n = 48) using conventional BC (p = n.s.). In six samples, multiplex-PCR detected more than one pathogen. Among the 47 microorganisms identified by SF, only 11 (23.4%) could be confirmed by BC. SF identified a significantly higher number of gram-negative bacteria than BC (28 vs. 11, χ^2^=7.97, p = 0.005). The combination of BC and SF significantly increased the number of detected microorganism compared to BC alone (48 (%) vs. 85 (%), χ^2^=13.51, p < 0.001). C-reactive protein (21.7 ± 11.41 vs. 16.0 ± 16.9 mg/dL, p = 0.009), procalcitonine (28.7 ± 70.9 vs. 11.5 ± 30.4 ng/dL, p = 0.015) as well as IL-6 (932.3 ± 1306.7 vs. 313.3 ± 686.6 pg/mL, p = 0.010) were significantly higher in patients with positive SF result. In addition, incidence of acute kidney injury requiring renal replacement therapy (31 (76%) vs. 125 (53%), p = 0.01) was higher in SF positive patients. Using ROC analysis, IL-6 (AUC 0.836, sensitivity 78.6%, specificity 75.9% for cut off 184 pg/mL) as well as C-reactive protein (AUC 0.804, sensitivity 71.4%, specificity 75.9% for cut-off 15.25 mg/dL) showed the best predictive values for positive SF results.

## Conclusions

The PCR-based SeptiFast test is a valuable addition to the traditional blood culture method to rapidly diagnose the etiology of bloodstream infections in patients after cardiothoracic surgery. This applies in particular for individuals with gram-negative bacteremia, organ failure and/or elevated CRP and IL-6-levels.

